# A simulated comparison of behavioural observation sampling methods

**DOI:** 10.1038/s41598-022-07169-5

**Published:** 2022-02-23

**Authors:** James Edward Brereton, Jonathan Tuke, Eduardo J. Fernandez

**Affiliations:** 1 University Centre Sparsholt, Sparsholt College, Westley Lane, Sparsholt, Winchester, SO21 2NF Hampshire UK; 2 School of Mathematical Sciences, The University of Adelaide, Adelaide, SA 5005 Australia; 3 School of Animal and Veterinary Sciences, The University of Adelaide, Adelaide, SA 5005 Australia

**Keywords:** Behavioural methods, Psychophysics

## Abstract

Behavioural research requires the use of sampling methods to document the occurrence of responses observed. Sampling/recording methods include ad libitum, continuous, pinpoint (instantaneous), and one-zero (interval) sampling. Researchers have questioned the utility of each sampling method under different contexts. Our study compared computerized simulations of both pinpoint and one-zero sampling to continuous recordings. Two separate computer simulations were generated, one for response frequency and one for response duration, with three different response frequencies (high, medium, or low) and response durations (short, medium, and long) in each simulation, respectively. Similarly, three different observation intervals (5, 50, and 500 s) were used to record responses as both pinpoint and one-zero sampling methods in the simulations. Under both simulations, pinpoint sampling outperformed one-zero sampling, with pinpoint sampling producing less statistical bias in error rates under all frequencies, durations, and observation intervals. As observation intervals increased, both mean error rates and variability in error rates increased for one-zero sampling, while only variability in error rate increased for pinpoint sampling. The results suggest that pinpoint sampling techniques are effective for measuring both frequency (event) and duration (state) behaviours, and that pinpoint sampling is a less statistically biased behavioural observation method than one-zero sampling.

## Introduction

Behavioural studies are a valuable tool for the scientific study of both human and non-human animal performance. In animal research, behavioural observations are used more often than other welfare indicators such as glucocorticoid analysis^1,2^. Behavioural research may also be used to investigate the prevalence of positive behaviours, like foraging, or negative behaviours, such as stereotypies^3–5^. Studies of behaviour are also frequently conducted for wild animal populations and to better understand natural histories or investigate the impact of human disturbance^2,6^. Research on animal behaviour is now so well recognised that there are numerous journals dedicated to its study, for instance: *Animal Behaviour*, *Applied Animal Behaviour Science*, and* Ethology.*

The methods used in behavioural research can be traced back to laboratory studies. Scientists during the mid-twentieth Century often used a mixture of both human and animal models to answer questions in the field of behavioural psychology^7,8^. Based on the range of different techniques that were generated by earlier studies, Altmann^9^ summarised the methods available. This paper became fundamentally important to those interested in behavioural research and remains a keystone paper for researchers. Other authors, such as Bateson and Martin^10^ further refined the behavioural methods and their definitions. Bateson and Martin^10^ distinguish between sampling and recording rules, which detail differences between the number of subjects observed (i.e. sampling rules; focal [1 subject] vs. scan [> 1 subjects]), or the observation method used to sample behaviour (i.e. recording rules; see below). However, little distinction is made between sampling and recording rules outside of this text, and for the purposes of our paper, we will refer to recording rules as sampling methods or techniques.

### Types of sampling (recording) methods

Since Altmann’s^9^ review, some behavioural sampling techniques have become increasingly popular in the research literature, whereas others are rarely used. Several behaviour measurement techniques have received criticism in terms of their repeatability^11^. For example, ad libitum (qualitative) sampling may be useful for developing ethograms and for pilot studies but has methodological flaws with regards to its lack of standardisation^12,13^. However*, *ad libitum sampling is still used in studies of behaviour, with a review by Mann^14^ identifying that between 53 and 59% of cetacean studies published in *Marine Mammal Science* used this sampling technique.

Continuous recording is considered the gold standard for behavioural sampling, as this method records all occurrences of behaviour and their durations^6,15^. In the past, this made continuous recording often challenging for researchers: for instance, an active animal that rapidly changed behaviour would have been difficult to observe and record^16^. Similarly, measurement of multiple animals using a continuous method would have been incredibly challenging to document accurately, hence the method is often considered synonymous with the focal sampling of one individual^9,12^. Use of modern technology has in part ameliorated some of these issues by allowing behaviour to be recorded and analysed later^17^. However, continuous recording may remain a challenge, especially where large amounts of data are being recorded or direct comparisons of response frequencies and durations are made, and as such there is a need for alternative methods. As a result, several sampling (recording) methods have been developed that allow multiple animals and behaviours to be measured at one time (scan sampling), as well in a non-continuous fashion.

The use of pinpoint sampling, also referred to as instantaneous or momentary time sampling, is a commonly used method for observational study^6,18–20^. With pinpoint sampling, one or more responses are recorded at preselected moments in time (e.g., every 15 s for an hour). The benefits of pinpoint sampling are that it is less intensive than continuous sampling, and therefore may be more feasible for researchers to conduct^12,21,22^. The methods are also more versatile, allowing researchers to make decisions as to how long intervals should be spaced. For example, some researchers might choose to use 15-s intervals, particularly when studying an active animal or when conducting observations of key times, such as when enrichment is provided^23,24^. On the other hand, observers might choose to use much longer intervals, such as one-, two- or five-minute intervals when their subjects are inactive or if they are observing for long time periods^25,26^. Shorter intervals tend to result in values that match more closely the continuous behaviour scores but require more recording effort^27^.

One-zero or interval sampling involves choosing specific intervals of time, like pinpoint sampling, but instead recording whether one or more responses occur (or conversely, do not occur) within that interval of time^6,28,29^. While popular with both human and non-human primate research, one-zero sampling seems to receive less representation than pinpoint sampling in most animal behaviour studies and has been criticised by previous researchers^9,30^. However, one-zero sampling has some of the same benefits of instantaneous sampling in that interval length can be tailored in line with the requirements of the study. Additionally, one-zero sampling has the potential to collect more behaviours during a predefined period, as multiple behaviours can be recorded during each interval^9^. Leger^31^ identified good agreement with continuous behaviour measures when using one-zero sampling at 15-s intervals for chimpanzees (*Pan troglodytes*). Likewise, Rhine and Flanigon^32^ found similar levels of occurrence when comparing continuous, pinpoint, and one-zero sampling methods with a colony group of stumptail macaques (*Macaca arctoides*). As noted above, one-zero (interval) sampling is also frequently used in studies on human behaviour, for example in the classroom^33,34^.

Both pinpoint and one-zero sampling overcome some of the issues associated with continuous recording by reducing the amount of input required by the researcher, while still aiming to keep the sample representative of the subject’s behavioural repertoire^35–38^. However, one key question is how closely these techniques correlate with continuous recording? A major concern focuses on distinguishing between the frequency vs duration of some response, with behaviours of short duration typically referred to as “events”, while behaviours of long durations are called “states”. Pinpoint sampling loses information in terms of the duration of any response and is potentially less likely to pick up any behaviours of short duration (events)^12,39^. By contrast, one-zero sampling is better at recording all observable behaviours, but both behavioural frequency and duration could be easily misrepresented: there is no way to identify whether a behaviour recorded as present for one interval was seen once or thirty times during that time period^40^.

### Sampling method simulations

Researchers in various fields have compared differences between pinpoint and one-zero sampling methods. Early simulations lacked the precision and/or ability to run extensive repetitions of their simulations to accurately assess sampling method differences^41–44^. Other researchers have attempted to make similar methodological comparisons via the data collection of actual behavioural occurrences^31,32,45–49^. While the results of differences in sampling methods for real occurrences of behaviour varied, most studies found pinpoint sampling to be more accurate than one-zero sampling, at least with respect to duration (state) behaviours. Nonetheless, caution should be used in making determinations of the validity of any result based on specific examples, as exceptions to any rule can and do occur.

Only three recent studies, all conducted by behaviour analysts interested in observations for applied, behaviour change purposes with human populations, have attempted to simulate data sets and compare some aspect of pinpoint and one-zero sampling methods^50–52^. In two of these studies^50,51^, limited simulations were produced via the rolling of die and pinpoint sampling was compared to a type of one-zero sampling, Partial Interval Recording (PIR), in which the response only need occur at any point during an observation interval to be recorded. In both studies, pinpoint sampling generally outperformed one-zero sampling for the detection of duration responses, with some variation in the ability of PIR to accurately detect frequency responses compared to pinpoint sampling and continuous recordings. Wirth et al.^52^ is the only study to date to use extensive computer-generated simulations to examine differences between pinpoint and one-zero sampling methods. Their study utilised both PIR and Whole Interval Recording (WIR), where the duration response must occur during the entire observation interval to be recorded. Overall, they found that pinpoint sampling outperformed one-zero sampling methods on most measures.

The following study proposes to compare computer simulated occurrences of both low/short, medium, and high/long frequency/duration behaviours, as well as similar observation intervals for pinpoint and one-zero sampling methods. Different durations of behaviour were used to provide generalised situations researchers may encounter: some behaviours are normally short (e.g. sneezing), medium (e.g. feeding) or long (e.g. resting) in their duration. We hypothesised that: (1) one-zero sampling would be more accurate (less statistically biased) for detecting the occurrence of low frequency (event) behaviours, particularly when comparing less frequent pinpoint and one-zero observation methods (e.g., 500 s observation intervals), and (2) pinpoint sampling would provide a more accurate representation of percentages of occurrence for both low, medium, and high duration (state) behaviours than one-zero sampling.

## Results

### Response frequency

**Figure 1:**
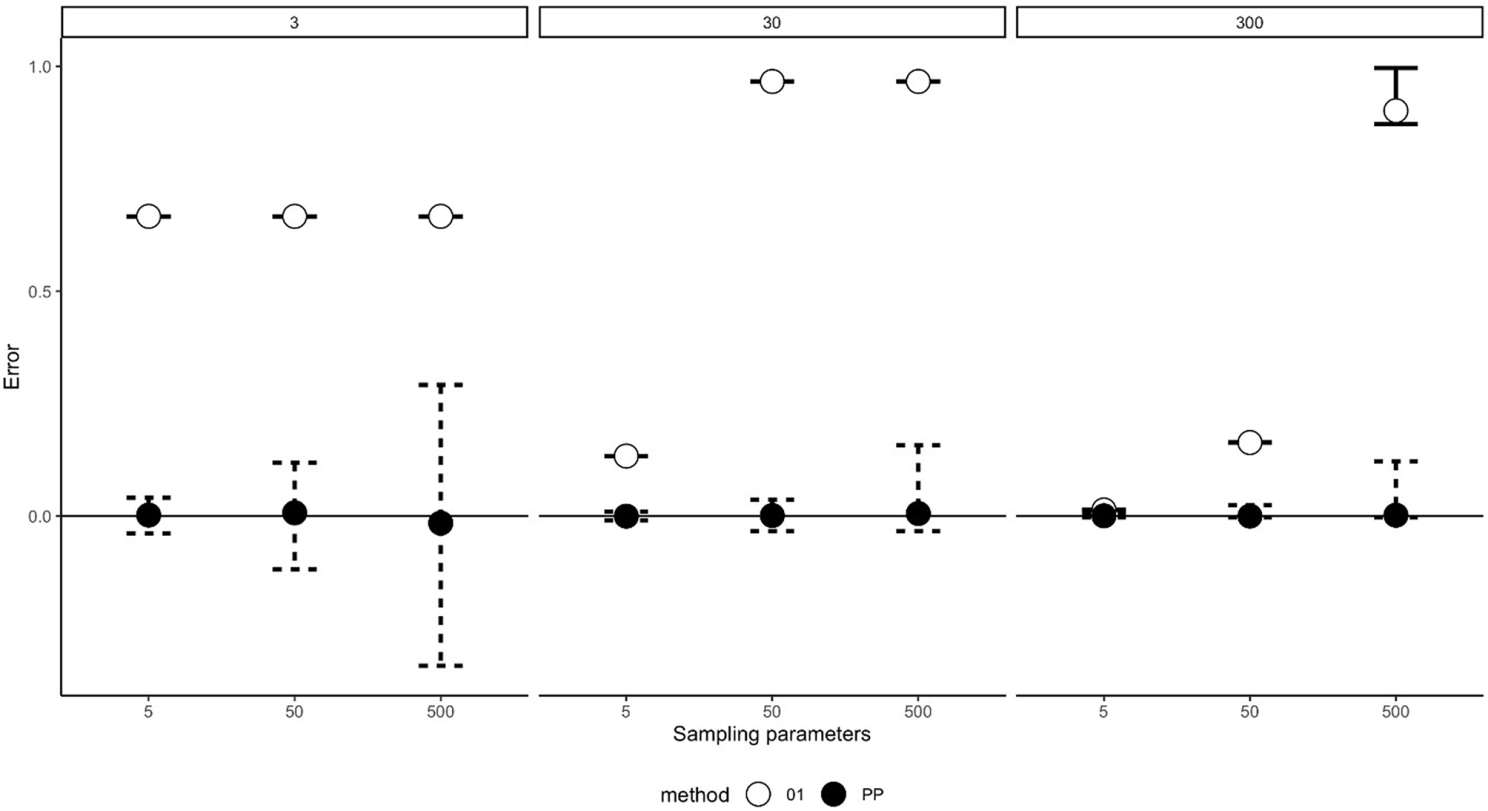
Mean error rates (with 95% percentile intervals of the errors bars) for the response frequency simulation and the pinpoint and one-zero sampling methods for the 5 s, 50 s and 500 s observation intervals. *PP* Pinpoint sampling, closed circles, *01* One-zero sampling, open circles.

The mean error rate for both pinpoint and one-zero sampling was calculated for each interval length and each of the three behavioural frequencies (see Fig. 1).

The mean error for pinpoint sampling was minimal for all interval lengths and behavioural frequencies. However, variance for the pinpoint sampling increased as interval length increased. For one-zero sampling, error rates increased as the interval length increased, with the 500 s interval showing the largest error rates irrespective of behavioural frequency.

Overall, mean error rates were consistently lower for the pinpoint sampling method in comparison to the one-zero sampling method (χ^2^ = 9, *df* = 1, *p* = 0.0027, *W* = 1) (see Table 1). Post-hoc tests for all 9 comparisons (3 frequencies × 3 recording intervals) were *p* < 0.001.

**Table 1:** Mean error rates for each sampling method under 5 s, 50 s and 500 interval lengths for the response frequency simulation.

Simulation parameters	Sampling parameters	Proportion of time event occurs	Mean error	Lower 95% percentile of error	Upper 95% percentile of error
**One-zero**					
3	5	0.333	0.667	0.667	0.667
3	50	0.333	0.667	0.667	0.667
3	500	0.333	0.667	0.667	0.667
30	5	0.033	0.133	0.133	0.133
30	50	0.033	0.967	0.967	0.967
30	500	0.033	0.967	0.967	0.967
300	5	0.003	0.013	0.013	0.013
300	50	0.003	0.163	0.163	0.163
300	500	0.003	0.902	0.872	0.997
**Pinpoint**					
3	5	0.333	0.002	− 0.039	0.0403
3	50	0.333	0.007	− 0.118	0.118
3	500	0.333	− 0.0158	− 0.003	0.292
30	5	0.033	− 0.0004	− 0.010	0.010
30	50	0.033	0.0010	− 0.033	0.036
30	500	0.033	0.005	− 0.033	0.157
300	5	0.003	0.0003	− 0.003	0.005
300	50	0.003	− 0.0003	− 0.003	0.024
300	500	0.003	0.0017	− 0.003	0.122

### Response duration

**Figure 2:**
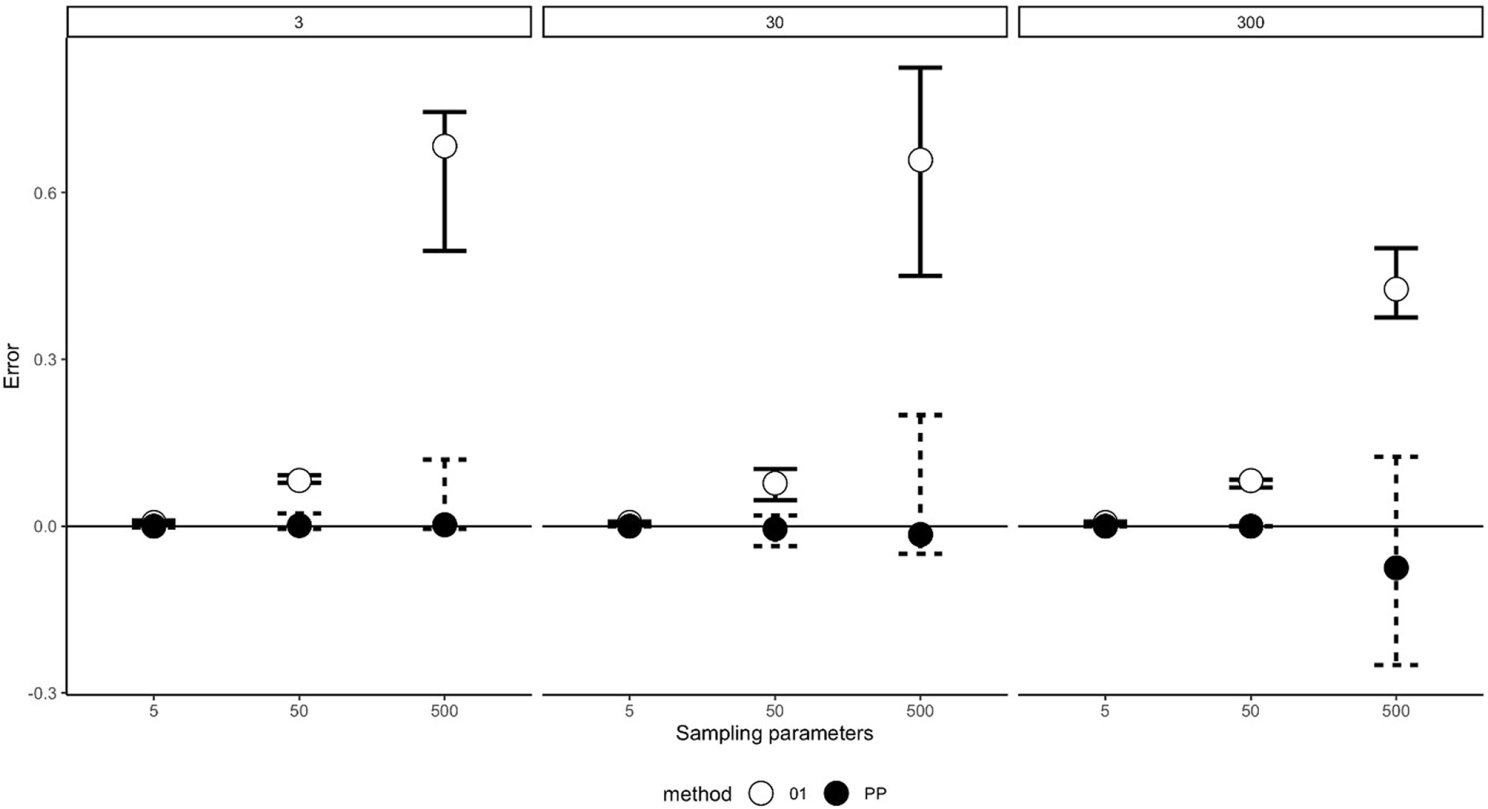
Mean error rates (with 95% percentile intervals of the errors bars) for the response duration simulation and the one-zero and pinpoint sampling methods for the 5 s, 50 s and 500 s observation intervals. *PP* Pinpoint sampling, closed circles, *01* One-zero sampling, open circles.

The accuracy of both pinpoint and one-zero sampling was calculated for each interval length and all three behavioural durations (short, medium, and long) (see Fig. 2).

For all simulation frequencies, pinpoint sampling was less statistically biased, with minimal error rates. By contrast, mean error rates were much higher for one-zero sampling, and these increased as interval length increased. For both pinpoint and one-zero sampling, the variance in error increased with interval length.

The pinpoint sampling method consistently produced lower error rates than the one-zero method (χ^2^  = 9, *df* = 1, *p* = 0.0027, *W* = 1) (see Table 2). Post-hoc tests for all 9 comparisons (3 durations × 3 recording intervals) were *p* < 0.001.

**Table 2:** Mean error rates for each sampling method under 5 s, 50 s and 500 interval lengths for the response duration simulation.

Simulation parameters	Sampling parameters	Proportion of time event occurs	Mean error	Lower 95% percentile of error	Upper 95% percentile of error
**One-zero**					
3	5	0.005	0.006	0.003	0.010
3	50	0.005	0.082	0.078	0.092
3	500	0.005	0.684	0.495	0.745
30	5	0.050	0.007	0.004	0.008
30	50	0.050	0.008	0.047	0.103
30	500	0.050	0.659	0.450	0.825
300	5	0.500	0.007	0.003	0.008
300	50	0.500	0.082	0.069	0.083
300	500	0.500	0.426	0.375	0.500
**Pinpoint**					
3	5	0.005	< 0.001	− 0.002	0.003
3	50	0.005	0.001	− 0.005	0.023
3	500	0.005	0.002	− 0.005	0.120
30	5	0.050	< 0.001	< 0.001	< 0.001
30	50	0.050	− 0.004	− 0.036	0.019
30	500	0.050	− 0.015	− 0.050	0.200
300	5	0.500	< 0.001	< 0.001	< 0.001
300	50	0.500	< 0.001	< 0.001	< 0.001
300	500	0.500	− 0.075	− 0.250	0.125

## Discussion

Our study attempted to answer two hypotheses: (1) one-zero sampling would be more accurate (less statistical error or bias) for detecting the occurrence of low frequency (event) behaviours, particularly when comparing less frequent pinpoint and one-zero observation methods, and (2) pinpoint sampling would provide a more accurate representation of percentages of occurrence for both low, medium, and high duration (state) behaviours than one-zero sampling. The first hypothesis was not supported, as pinpoint sampling was better able to detect frequency responses than one-zero sampling, even when events occurred less frequently, and when recording intervals were longer. The second hypothesis was supported in that pinpoint sampling had lower error margins than one-zero sampling for detecting duration behaviours. One-zero sampling was similarly capable at detecting duration behaviours of any length at low (5 s) or medium (50 s) recording intervals. At longer recording intervals (500 s), pinpoint sampling substantially outperformed one-zero sampling for the detection of duration (state) behaviours. Finally, for both sampling methods, increasing the interval recording length appeared to increase the variability in error rates for both frequency and duration responses. As the recording interval increased, one-zero sampling became less accurate (more statistically biased), as observed by an increase in mean error rate. Increased recording intervals also increased variability in the mean error rate for one-zero sampling of duration responses. Pinpoint sampling maintained low error rates regardless of the recording interval length, however, as the recording interval increased, pinpoint sampling showed greater variability in the mean error rate for both frequency and duration responses.

As noted in the Introduction, Wirth et al.^52^ is the only other study to date to use extensive computer-generated simulations to examine differences between pinpoint and one-zero sampling methods, in their case both partial interval recording (PIR) and whole interval recording (WIR) methods. Like our study, they generated 100 simulations, and found pinpoint sampling to be more accurate (less statistically biased) than PIR or WIR, which overestimated and underestimated cumulative event durations, respectively. One limitation of their simulation was that it used a truly randomized rather than block structure for the simulated responses, as ours did, which more directly limits the applicability of their simulation to real-world behaviours (behaviour is rarely, if ever, truly random). Regardless, their results were similar to our study in that pinpoint sampling was generally more accurate than one-zero sampling methods.

Taken together, the results of our study and previous simulations suggest that pinpoint sampling is more accurate in detecting responses than one-zero sampling. Below we consider these implications, as well as factors that should influence the selection of behavioural sampling methods.

### Which sampling method is most appropriate for my study?

Pinpoint sampling has not been recommended for measuring frequency (event) responses, particularly those of low occurrence^6,9^. However, in our simulation this method was accurately able to detect low occurrence (< 1%) frequencies. Therefore, the use of pinpoint sampling to measure any event responses, regardless of their frequency of occurrence, appears to be a viable option if large amounts of behavioural data are collected.

One-zero sampling methods are often preferred as an observational method because of the ease with which behaviours can be observed, recorded, and assessed for Interobserver Agreement (IOA)^53,54^. The same can also be said for pinpoint sampling, which provides an equally user-friendly research method when compared to continuous (focal) recordings. In addition, researchers attempting to account for under- or over-estimates of one-zero recordings have devised different sampling methods, including partial, whole, occurrence, and non-occurrence interval (one-zero) recordings. Still, the difficulty here is that, if pinpoint sampling provides a more accurate representation of behavioural occurrence, then the solution should be to adopt this method rather than adjusting to a less accurate one-zero recording method.

An added benefit of using either pinpoint or one-zero sampling methods over continuous recordings are that frequency (event) versus duration (state) behaviours can be compared more clearly. For instance, if a researcher were assessing the impact of pacing on the welfare of an animal, measuring pacing as an event or state would result in different data being generated. Lehner^6^ suggests that the former could be assessed as a bout of event responses, but it is still not clear how to evaluate the difference between about of responses to less frequent but longer duration behaviours. Pinpoint and one-zero sampling methods avoid this problem by only recording whether the response occurred during some observation period, regardless of the frequency or duration of the recorded response. This makes these observation methods valuable in circumstances where presence or absence of a particular behaviour is more important than the measurement of its frequency or duration, such as in studies of courtship or reproduction^10,12^.

There may remain several valuable uses for one-zero sampling as a tool for researchers. For example, one-zero sampling may still be the most useful technique when a specific, important behaviour occurs very rarely and is of short duration. The value of one-zero sampling would be further enhanced in studies where smaller amounts of data are collected. Examples could include courtship displays, where the behaviours may occur only a handful of times per individual per year for some species^12^. The chance of the behaviour being recorded by pinpoint sampling may be minimal, yet the value of identifying the behaviour may be disproportionately high. However, caution is still warranted in the application of one-zero sampling methods to record rare, short duration responses, as it is not clear whether such interval recording methods would produce an accurate representation of such low occurrence responses.

### Sampling method selection and laboratory lore

Historically, a major factor in determining behavioural observation methodology has been the prevalence of that sampling method within some field or observational species. For instance, Mann^14^ found that over half of all cetacean studies in their review used ad libitum sampling, even though such sampling methods are recognized to be both less quantitative and systematic. Likewise, one-zero sampling methods are typically used by primatologists and behaviour analysts for the study of non-human primate and human behaviour, respectively^30–36,49,53–57^. The concept of using methodology passed down from previous studies and labs has been referred to as “laboratory lore” and is an asset to the cultural transmission of scientific knowledge^58,59^. Nonetheless, the selection of behavioural observation methods, like all aspects of scientific research, should be based on the efficacy of the methodology used. In the case of selecting between pinpoint or one-zero sampling methods to estimate behavioural occurrences, our study indicates that pinpoint sampling outperforms one-zero sampling on all frequency (event) and duration (state) measures simulated. Thus, laboratory lore aside, pinpoint sampling seems to be the better option for measuring some aspect of behavioural prevalence when compared to one-zero sampling methods.

## Methods

For all simulations, patterns of behaviour were computer generated for both frequency of occurrence (how often the behaviour appeared) and percentage of occurrence (the percentage of time that the behaviour occurred). On these simulated patterns of behaviour, two different non-continuous sampling methods were directly compared: pinpoint (instantaneous) and one-zero (interval) sampling. Two sets of simulations were produced: response frequency (to measure the ability of both behaviour methods to detect short, event behaviours at different rates of occurrence) and response duration (to measure the ability of the methods in assessing state behaviours of different lengths). Three levels for response frequency and response duration were determined, based on a level of frequency/duration: 3 s, 30 s, and 300 s. These three durations were selected because they are reflective of different durations of behaviour in published studies^10,12^. The interval lengths for both pinpoint and one-zero sampling were set at 5 s, 50 s, and 500 s, in order to compare the effect of interval length on sample accuracy. These three interval lengths were chosen to reflect some of the common sampling lengths (frequent, regular and infrequent) used in human and animal research^10,12^.

### Simulations

All the simulations were done in the R computing language version 3.6.3 using the GUI RStudio (code publicly available at https://github.com/jonotuke/animal_simulation_2020)^60^. For both sets of simulations, observation periods were set to a length of one hour, or 3600 s, as this time length is often set in observational studies^61^,. A total of 1800 h of simulated data were generated across the response frequency and duration conditions.

### Response frequency

**Figure 3:**
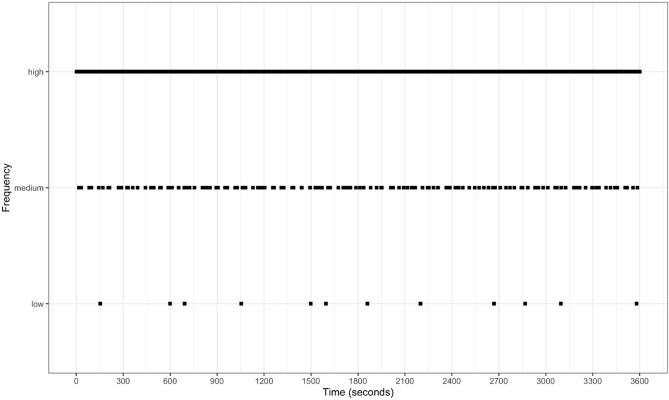
Example of simulated data for response frequency for high (3 s), medium (30 s), and low (300 s) frequency behaviours. This results in the frequency (event) occurring for exactly 33, 3.3, and 0.3% of the one-hour simulation, respectively. The high frequency occurs so often that it appears as a solid line, but the behaviour is in fact occurring once in each 3 s period.

This simulation focused on the recording of event behaviours: behaviours of short duration^10^. For the simulation, the duration of all event behaviours was set to exactly one second. Next, three different frequencies of event behaviour were selected: high (3 s), medium (30 s) and low (300 s) frequency of occurrence, in order to reflect different types of behaviour that occur very frequently, less frequently, or infrequently^62^ (Fig. 3). The observation period was one-hour in length (3600 s). A total of 100 simulated data sets were generated for each of the three response frequencies. The exact time that each event occurred within the 3, 30 or 300 s period was randomised within the predefined blocks (e.g. the behaviour exactly once within its 3, 30 or 300 s period).

The real (continuous) occurrence of each simulated response frequency was determined by calculating the number of seconds of each event that were possible in a simulated hour of data (observation period divided by frequency of occurrence; high frequency = 1200 s; medium frequency = 120 s; and low frequency = 12 s). The event behaviour seconds were then transformed into a percentage of total time (as is often shown in behaviour studies in the form of an activity budget), as well as frequency of occurrence. Thus, high frequency (3 s) responses occurred 33% of the hour, medium frequency (30 s) responses occurred 3.3%, and the low frequency (300 s) responses occurred 0.3% of the time.

To compare against this real (continuous) measurement, pinpoint and one-zero sampling were used on the simulated data sets. One-zero sampling recorded an event if it occurred at any point during the observation period, also commonly referred to as partial interval recording (PIR). The three interval lengths (5, 50, and 500 s) were used for both pinpoint and one-zero sampling. This resulted in nine-hundred data sets (nine combinations of simulation parameters and sampling parameters, each combination simulated 100 times) being developed.

### Response duration

**Figure 4:**
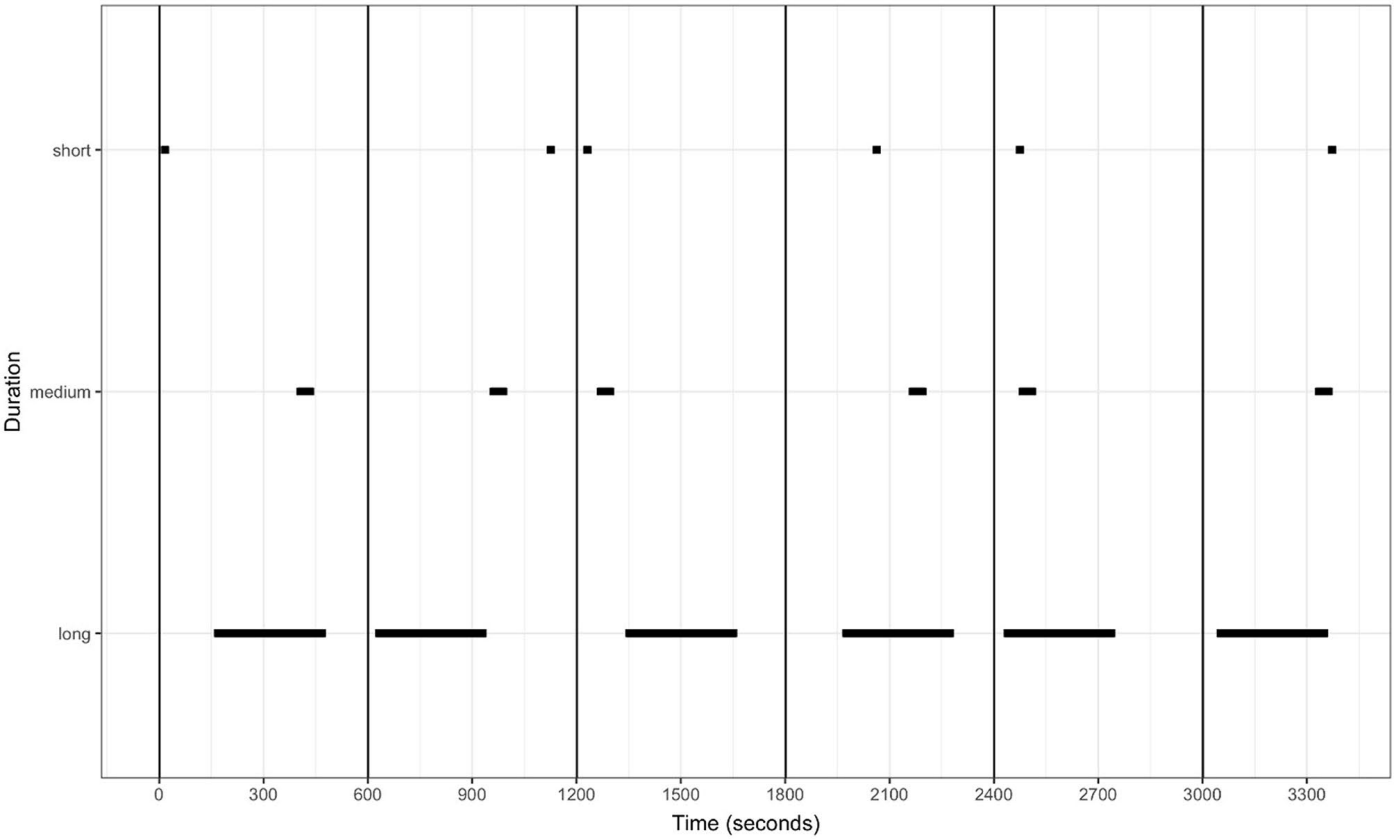
Example of simulated data for response duration for short (3 s), medium (30 s), and long (300 s) duration behaviours. The location of each state was selected at random within its 600 s period. This results in the duration (state) lasting for exactly 0.5, 5, and 50% of the one-hour observation in total respectively.

This simulation was developed for longer duration or state behaviours. In the literature, state behaviours can be of variable length, lasting anywhere from seconds (e.g. scratching) to minutes (e.g. preening) or hours (e.g. resting). To accommodate this, three levels of behavioural duration were selected. These durations were set as short (3 s), medium (30 s) and long (300 s) durations of occurrence (Fig. 4). Each of these states were treated separately (only short, medium, or long behaviours occurred in each simulation). As per the *Response Frequency* investigation, the observation period was set to one-hour in length (3600 s). Each duration simulation was repeated 100 times.

The chosen behaviour occurred once per 600 s period. The exact time that each behaviour occurred within its respective 600 s period was selected at random (though the behaviour was not allowed to slip into the next period of 600 s). Continuous data sets were developed by using the raw, simulated data and transforming this into percentages. This meant that each behaviour occurred six times during each one-hour simulation, with the short duration (3 s) responses occurring 0.5% of the hour, the medium duration (30 s) responses occurring 5%, and the long duration (300 s) responses occurring 50% of the time.

Each of the three behaviour durations (short, medium, and long) were measured using one-zero (PIR) and pinpoint sampling. Three interval lengths were recorded, again consisting of 5 s, 50 s and 500 s, as had been selected for the *Response Frequency* investigations. These interval lengths were used for both the pinpoint and one-zero sampling. Once complete, the results were then transformed into percentages and compared to the continuous data to determine the level of error.

### Statistical analysis

Statistical analyses were conducted on the mean error scores for pinpoint and one-zero sampling at each respective interval length. The Friedman test was used to investigate whether there was a statistically significant effect of sampling method on the estimation error. The sampling/simulation combination was used as a blocking factor. The non-parametric Friedman test was used due to the non-normality of the errors and the observed heteroscedascity. When significant differences were found, paired Wilcoxon tests were used to compare the treatments. To compensate for multiple comparisons, we used an FDR adjustment. The method is statistically not-biased, meaning the process is giving an estimate of the true parameters that are correct (i.e. not over- nor under-estimated).
